# Detectable Changes in The Blood Transcriptome Are Present after Two Weeks of Antituberculosis Therapy

**DOI:** 10.1371/journal.pone.0046191

**Published:** 2012-10-02

**Authors:** Chloe I. Bloom, Christine M. Graham, Matthew P. R. Berry, Katalin A. Wilkinson, Tolu Oni, Fotini Rozakeas, Zhaohui Xu, Jose Rossello-Urgell, Damien Chaussabel, Jacques Banchereau, Virginia Pascual, Marc Lipman, Robert J. Wilkinson, Anne O’Garra

**Affiliations:** 1 Division of Immunoregulation, MRC National Institute for Medical Research, London, United Kingdom; 2 Division of Mycobacterial Research, MRC National Institute for Medical Research, London, United Kingdom; 3 Department of Respiratory Medicine, Imperial College Healthcare NHS Trust, London, United Kingdom; 4 Institute of Infectious Diseases and Molecular Medicine, University of Cape Town, Cape Town, South Africa; 5 Department of Medicine, Wright Fleming Institute, Imperial College, London, United Kingdom; 6 Baylor Institute for Immunology Research/ANRS Center for Human Vaccines, INSERM, Dallas, Texas, United States of America; 7 Systems Immunology, Benaroya Research Institute, Seattle, Washington, United States of America; 8 Department of Thoracic Medicine, Royal Free Hospital NHS Trust, London, United Kingdom; Statens Serum Institute, Denmark

## Abstract

**Rationale:**

Globally there are approximately 9 million new active tuberculosis cases and 1.4 million deaths annually**.** Effective antituberculosis treatment monitoring is difficult as there are no existing biomarkers of poor adherence or inadequate treatment earlier than 2 months after treatment initiation. Inadequate treatment leads to worsening disease, disease transmission and drug resistance.

**Objectives:**

To determine if blood transcriptional signatures change in response to antituberculosis treatment and could act as early biomarkers of a successful response.

**Methods:**

Blood transcriptional profiles of untreated active tuberculosis patients in South Africa were analysed before, during (2 weeks and 2 months), at the end of (6 months) and after (12 months) antituberculosis treatment, and compared to individuals with latent tuberculosis. An active-tuberculosis transcriptional signature and a specific treatment-response transcriptional signature were derived. The specific treatment response transcriptional signature was tested in two independent cohorts. Two quantitative scoring algorithms were applied to measure the changes in the transcriptional response. The most significantly represented pathways were determined using Ingenuity Pathway Analysis.

**Results:**

An active tuberculosis 664-transcript signature and a treatment specific 320-transcript signature significantly diminished after 2 weeks of treatment in all cohorts, and continued to diminish until 6 months. The transcriptional response to treatment could be individually measured in each patient.

**Conclusions:**

Significant changes in the transcriptional signatures measured by blood tests were readily detectable just 2 weeks after treatment initiation. These findings suggest that blood transcriptional signatures could be used as early surrogate biomarkers of successful treatment response.

## Introduction

Approximately one third of the world’s population is infected with the pathogen *Mycobacterium tuberculosis* (*Mtb)*, the cause of tuberculosis (TB). While most remain asymptomatic, with latent TB, approximately 10% develop active TB during their lifetime [1]. Over nine million new cases of active TB and 1.4 million deaths annually have been reported [2]. Improved diagnostics, treatments of shorter duration and improvements in treatment monitoring are badly needed.

Active pulmonary TB diagnosis requires culture of *Mtb*, which may take up to 6 weeks [3]. Although the World Health Organisation (WHO) endorsed GeneXpert MTB/RIF automated molecular test for *Mtb* results in rapid diagnosis [4], this test still requires sputum which may be difficult to obtain. Difficulties in obtaining sputum lead to approximately 30% of patients in the USA and 50% of South African patients to be treated empirically [2], [5]. After diagnosis there are no available early biomarkers correlating with treatment success, resulting in significant delay in assessing treatment response. Currently conversion to negative culture after 2 months of treatment is the only accepted biomarker [6]. However a systematic review and meta-analysis of sputum conversion revealed low sensitivity and modest specificity for the prediction of treatment failure [7]. Chest X-rays are commonly used to assess response but are not universally available and assessment is difficult to standardise [8]. This lack of effective treatment monitoring can lead to the development and spread of multidrug resistant (MDR) and extensively drug resistant (XDR) TB, which are mainly caused by non-adherence or inappropriate drug regimens, with a detrimental impact on global TB control.

To date transcriptional profiling has been used successfully in cancer classification, to identify prognostic biomarkers [9], and to distinguish between inflammatory and infectious diseases [10]. Moreover we recently demonstrated a whole blood transcriptional signature which could distinguish active TB from latent TB and other diseases, and which correlated with radiographic extent of disease [11]. This active TB blood signature diminished in seven patients after 2 months of successful treatment and reverted to that of healthy individuals after completing treatment [11]. Earlier blood biomarkers correlating with treatment response would improve monitoring of individual patient treatment responses without the need for sputum production, which may permit stratification of patients requiring differing treatment regimens. Additionally early biomarkers may aid in anti-TB drug development.

Our study was therefore designed to establish if early changes in blood transcriptional responses can be observed during standard anti-TB treatment. In addition this adds to our previous study by examining the transcriptional treatment response directly in a larger cohort from a high-burden TB country, South Africa [2]. Our study demonstrated that a change in the whole blood host transcriptional signatures was significantly detectable as early as 2 weeks after initiation of treatment, providing potential for the development of early biomarkers for treatment monitoring.

## Materials and Methods

### Study Population and Inclusion Criteria

**Figure 1:**
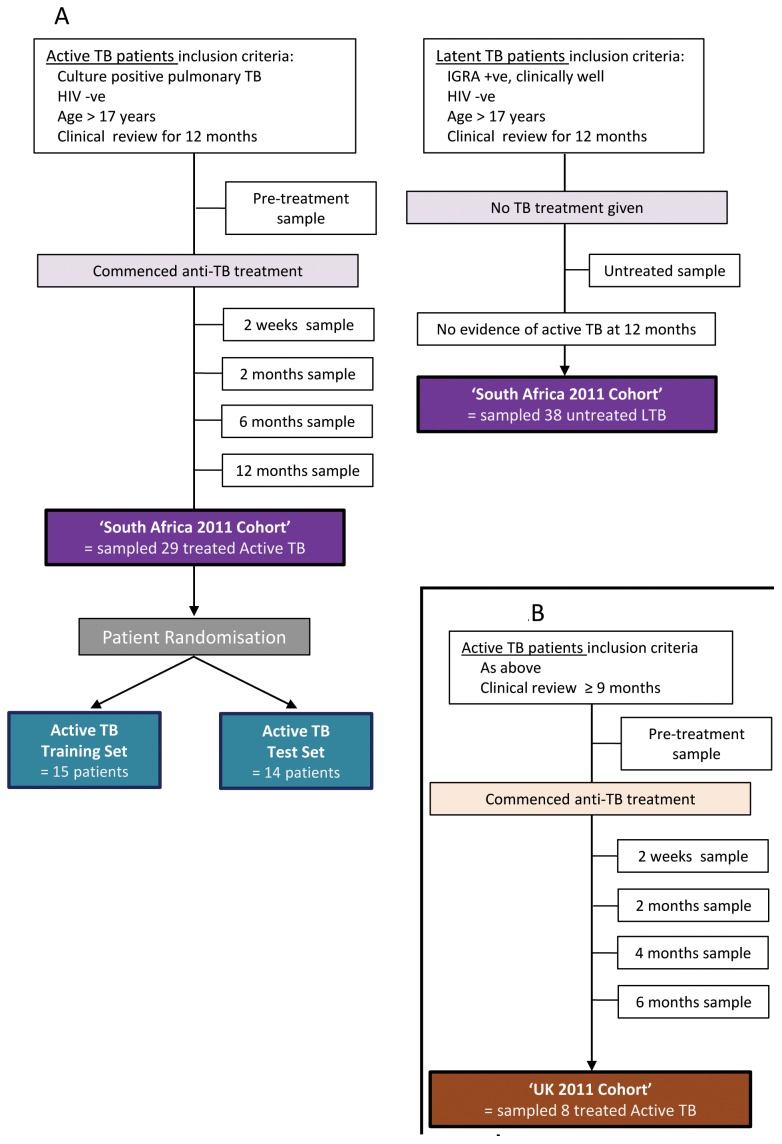
Numbers enrolled and assigned to cohorts. **(A)** South Africa: A total of 67 active and latent TB patients were enrolled into the untreated South Africa 2011 Cohort. A total of 29 active TB patients were included in the treated South Africa 2011 Cohort. 15 were randomised into the Active TB Training Set and 14 into the Active TB Test Set. **(B)** UK: A total of 8 active TB patients were enrolled into the treated UK 2011 Cohort.

All participants in South Africa were recruited from the Ubuntu TB/HIV clinic in Khayelitsha, a large peri-urban African township in Cape Town which has over 1000 TB notifications annually. During the period May 2008– August 2010 whole blood was collected from adult patients (age >17 years) with drug sensitive *Mtb* culture proven active pulmonary TB (Figure 1A). Due to the population’s high *Mtb* exposure, controls were considered as asymptomatic individuals with previous exposure to *Mtb* (latent TB patients); exposure was evidenced by a positive QuantiFERON-TB Gold In-Tube (QFT) (Cellestis). Participants with latent TB were recruited from individuals self-referring to the voluntary testing clinic. All participants had negative HIV status.

The UK 2011 Active TB Validation Cohort were all *Mtb* culture proven adults (>17 years) recruited between August 2009 - November 2011 from the Royal Free Hospital, London (Figure 1B). All participants in our earlier 2009 study were selected as previously described [11]. Clinical and demographic data was recorded for all participants and stored in a database.

### Ethics Statement

This study was approved by the University of Cape Town Faculty of Health Sciences Human Research Ethics Committee, Cape Town, South Africa (FHS HREC 012/2007), and the Central London 3 Research Ethics Committee (09/H0716/41). All participants gave informed written consent.

### Follow Up Period

All 29 treated 2011 South Africa active TB patients completed a full 6 months of treatment. Patients were sampled for venous blood at time points: pre-treatment (29/29 patients), 2 weeks (25/29 patients), 2 months (24/29 patients), 6 months (25/29 patients) and 12 months (29/29 patients) after initiation of treatment (Figure 1A). Patient’s response to anti-TB treatment was assessed clinically during the 12 month period. All patients were discharged from the program as cured.

Eight treated 2011 UK Active TB patients completed a full 6 months of treatment, one patient completed 9 months of treatment due to radiographic uncertainty of treatment success. Each patient was sampled for venous blood at 2 weeks, 2 months, 4 months and 6 months after initiation of treatment (Figure 1B). Four patients had a sample at every time point, three patients had samples at 2, 4 and 6 months, and four patients had samples at 2 weeks and 6 months. As part of their routine medical care all patients had chest X-rays at the beginning and end of their treatment.

### IFNγ Release Assay Testing

The QFT Assay (Cellestis) was performed according to the manufacturer’s instructions.

### Gene Expression Profiling

3 ml of whole blood were collected into Tempus tubes (Applied Biosystems/Ambion) by standard phlebotomy, vigorously mixed immediately after collection, and stored between −20 and −80°C before RNA extraction. South Africa and UK 2011 sample’s RNA was isolated using 1.5 ml whole blood and the MagMAX-96 Blood RNA Isolation Kit (Applied Biosystems/Ambion) according to the manufacturer’s instructions. 250 µg of isolated total RNA was globin reduced using the GLOBINclear 96-well format kit (Applied Biosystems/Ambion) according to the manufacturer’s instructions. Total and globin-reduced RNA integrity was assessed using an Agilent 2100 Bioanalyzer (Agilent Technologies). RNA yield was assessed using a NanoDrop800 spectrophotometer (NanoDrop Products, Thermo Fisher Scientific). Biotinylated, amplified antisense complementary RNA (cRNA) targets were then prepared from 200–250 ng of the globin-reduced RNA using the Illumina CustomPrep RNA amplification kit (Applied Biosystems/Ambion). 750 ng of labelled cRNA was hybridized overnight to Illumina Human HT-12 V4 BeadChip arrays (Illumina), which contained more than 47,000 probes. The arrays were washed, blocked, stained and scanned on an Illumina iScan, as per manufacturer’s instructions. GenomeStudio (Illumina) was then used to perform quality control and generate signal intensity values.

The 393- and 86-transcript signatures were translated from the HT-12 V3 BeadChip arrays to HT-12 V4 BeadChip arrays using GeneSpring GX version 11.5 (Agilent Technologies) and translated to slightly fewer probes in V4 (Figure S3).

Raw data were processed using GeneSpring GX version 11.5 (Agilent Technologies) and the following was applied to all analyses. After background subtraction each probe was attributed a flag to denote its signal intensity detection *p*-value. Flags were used to filter out probe sets that did not result in a ‘present’ call in at least 10% of the samples, where the ‘present’ lower cut off  = 0.99. Signal values were then set to a threshold level of 1, log2 transformed, and per-chip normalised using 75^th^ percentile shift algorithm. Next per-gene normalisation was applied by dividing each messenger RNA transcript by the median intensity of the latent TB samples. All statistical analysis was performed after this stage.

The raw and normalised microarray data has been deposited with the GEO (GSE40553). All data collected and analysed in the experiments adhere to the Minimal Information About a Microarray Experiment (MIAME) guidelines.

### Data Analysis

GeneSpring 11.5 was used to select transcripts that displayed a degree of expression variability. A filter was set to include only transcripts that had at least twofold changes from the median and present in at least 10% of the samples. To divide the South Africa 2011 cohort into a training and test set we used a computer algorithm for randomisation [12]. For the specific treatment response signature transcripts had to satisfy a threefold expression filter in 12 of the 15 training set matched untreated and 6 month treated samples.

**Figure 2:**
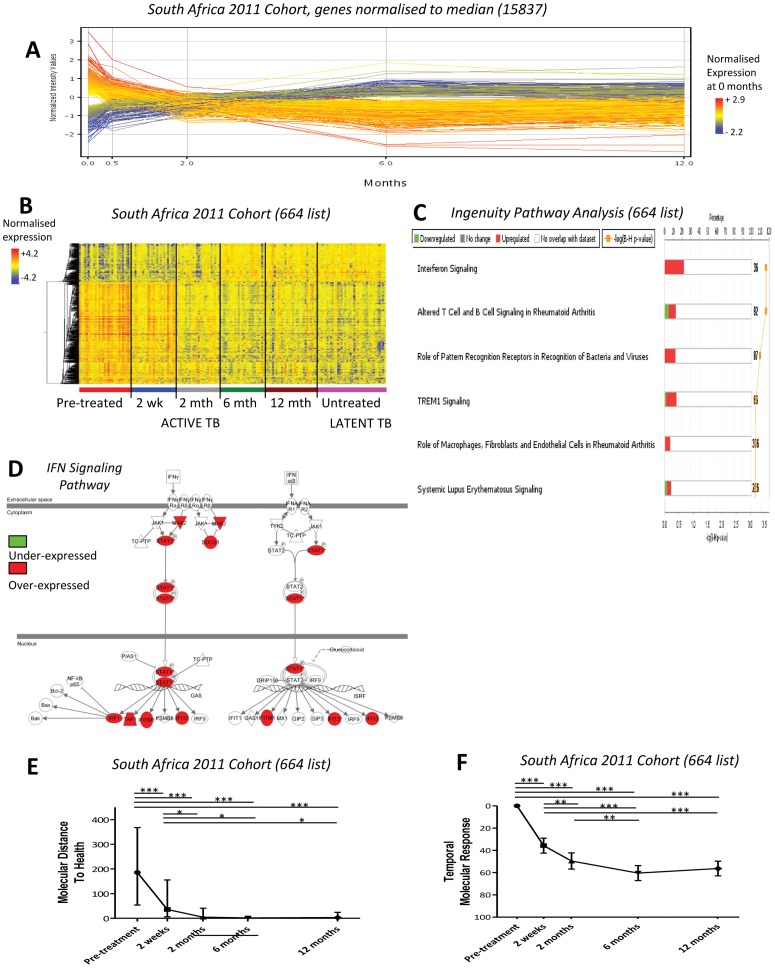
A blood transcriptional response is detectable after only 2 weeks of treatment. **(A)** Profile plot of all detectable transcripts (15837) obtained without any filtering, in the treated active TB patients in the South Africa 2011 cohort. It can be seen that gene expression changes after just 2 weeks of treatment. **(B)** 664 differentially expressed transcripts between untreated active and latent TB patients in the untreated South Africa 2011 cohort, were obtained by twofold change from the median and stringent statistical filtering (Mann Whitney, Bonferroni p<0.01). The heatmap shows the dynamic change of gene expression in response to treatment in the treated South Africa 2011 cohort normalised to the median of all transcripts. **(C)** Ingenuity Pathway Analysis (IPA) of the 664 transcripts shows the top significant pathways. **(D)** Interferon signaling pathway from the 664 list in IPA. **(E)** Weighted molecular distance to health (MDTH) of the treated South Africa 2011 cohort shows the signature significantly diminishes over time (linear mixed models, bars represent median & IQR, *** = p<0.001, ** = p<0.01, * = p<0.05). **(F)** Temporal molecular response further shows significant and early changes in response to anti-TB treatment (linear mixed models, bars represent mean & 95% confidence intervals).

Selected transcripts were then filtered by different levels of statistical stringency in GeneSpring 11.5. Non-parametric tests with multiple testing corrections were applied to all analyses [13], [14]. The active TB-transcriptional signatures was generated by Mann Whitney unpaired Bonferroni p<0.01 (Figure 2A). The statistical filter used to generate the specific TB treatment response-transcriptional signature was Mann Whitney paired Benjamini Hochberg p<0.01. The 393 and 86 active TB signatures were obtained as described previously (Figure S2) [11]. Visualisation of the data was performed by heatmaps using hierarchical clustering where the correlation distance metric employed for the clustering was Pearson’s uncentered with average linkage [15]. Heatmaps displayed either hierarchical clustering of both transcripts and samples or hierarchical clustering of transcripts with forced grouping of samples. Visualisation of common and different transcripts by venn diagrams was performed in GeneSpring 11.5.

Molecular distance to health (MDTH) was determined for each time point, as previously described [16]. The temporal molecular response was calculated for a particular gene list for each individual patient. The raw intensity transcript values in the gene list were consecutively compared at each time point to the baseline (pre-treatment). The numbers of transcripts that were at least two-fold up or two-fold down from the baseline were added together for each time point. This sum was then divided by the total number of transcripts in the gene list to calculate a percentage score for each time point. This generated a percentage score of change at each time point compared to the baseline, where the baseline always remains zero (no change from itself). To allow for two-fold changes from zero any baseline raw transcript intensity values of zero were converted to 10^−20^. MDTH and temporal molecular response were calculated in Microsoft Excel 2010. GraphPad Prism version 5 for Windows was used to generate graphs and determine simple linear regression. Linear mixed models, fixed effects, were used to determine *p*-values associated with MDTH and temporal molecular response graphs, using S*AS*/STAT®software (SAS Institute Inc., USA). Pathway analyses were performed using Ingenuity Pathway Analysis (Ingenuity Systems, Inc., Redwood, CA). Canonical pathways analysis identified the most significantly represented pathways in the datasets (Fisher’s exact Benjamini Hochberg p<0.05).

## Results

### Participants Demographics and Characteristics

Participant numbers in the 2011 cohorts are described in Figure 1; 29 South African and 8 UK active TB patients were recruited and sampled for transcriptomic analysis. All treated active TB patients had fully sensitive *Mtb*, took all treatment prescribed, showed successful clinical/radiological response to standard therapy (rifampin, pyrazinamide, isoniazid and ethambutol for 2 months followed by rifampin and isoniazid for 4 months), did not relapse within 1 year and were discharged from the program as cured. The 29 South African patients were sampled at: pre-treatment (29/29 patients), 2 weeks (25/29 patients), 2 months (24/29 patients), 6 months (25/29 patients) and 12 months (29/29 patients) after initiation of treatment. Thirty-eight South African latent individuals were sampled as asymptomatic controls. Only five latent individuals were aware of prolonged contact with another individual with active TB. Participant characteristics are reported in Table S1 in the online data supplement.

### A Change in Transcriptional Response is Readily Detectable after 2 Weeks of Treatment

To determine whether an active TB blood transcriptional signature was perturbed upon treatment, gene expression profiles of significantly detectable genes without further filtering (detected p<0.01 from background, 15,837 transcripts), were examined in the 29 active TB patients before, during (2 weeks and 2 months), at the end of (6 months), and after treatment (12 months). By plotting the expression profiles of the 15,837 transcripts along a time scaled x-axis, a marked change was readily observed after 2 weeks of anti-TB treatment (Figure 2A).

Next an active TB 664-transcript signature (Table S2) was derived from differentially expressed genes in the pre-treatment active TB patients compared to the latent TB patients in the South Africa 2011 cohort. First, all transcripts were normalised to the median of the latent TB patients, then only transcripts with ≥ twofold change from the median were selected, before applying a statistical filter. When this signature was applied to the South Africa 2011 Cohort, during and after treatment, a marked and rapid change in the transcriptional response was observed as early as 2 weeks, which then continued through 2 and 6 months, after treatment initiation (Figure 2B). In agreement with our previous study, Ingenuity Pathway Analysis (IPA) of the active TB 664-transcript signature demonstrated a highly significant over-representation of Interferon (IFN)-signaling genes including Type I and Type II IFN (Figure 2C and D, p<0.001, Table 1).

**Table 1:** Genes present in the top Ingenuity Pathway Analysis pathways in the active TB 664-transcript signature.

Interferon Signaling		Altered T & B cell Signaling In Rheumatoid arthritis		Role of Pattern Recognition Receptors in Recognition of Viruses & Bacteria		TREM1 Signaling		Role of Macrophages, Fibroblasts, Endothelial cells in Rheumatoid Arthritis		Systemic Lupus Erythematous Signaling	
Gene	FC	Gene	FC	Gene	FC	Gene	FC	Gene	FC	Gene	FC
IFI35	2.32	CD40LG	−3.09	C5	2.45	CASP1	2.11	C5	2.45	C5	2.45
IFIT3	5.81	CD79A	−5.09	C1QB	24.63	CASP5	8.58	CREB5	3.70	CD3E	−2.20
IFITM1	2.39	CD79B	−2.15	C1QC	4.66	IL1B	2.29	F2RL1	2.27	CD40LG	−3.09
IRF1	2.26	FAS	2.43	CASP1	2.11	ITGAX	4.97	FCGR1A	10.37	CD79A	−5.09
JAK2	2.38	FCER1G	2.05	IFIH1	2.39	JAK2	2.38	IL15	5.53	CD79B	−2.15
SOCS1	3.33	IL15	5.53	IL1B	2.29	NOD2	2.50	IL18R1	2.41	FCER1G	2.05
STAT1	3.40	IL1B	2.29	IRF7	2.25	PLCG1	−2.02	IL18RAP	2.32	FCGR1A	10.37
TAP1	2.42	IL1RN	2.06	NLRC4	3.17	TLR2	3.12	IL1B	2.29	FCGR1B	12.33
		SLAMF1	−2.84	NOD2	2.50	TLR5	3.07	IL1RN	2.06	FCGR1C	9.66
		TLR2	3.12	TLR2	3.12			IRAK3	2.05	FCGR2C	5.03
		TLR5	3.07	TLR5	3.07			JAK2	2.38	FCGR3B	2.13
		TNFSF13B	2.36					MAPK14	5.04	IL1B	2.29
								NFAT5	2.56	IL1RN	2.06
								OSM	2.76	LCK	−2.22
								PDGFA	2.16	NFAT5	2.56
								PLCG1	−2.02	PLCG1	−2.02
								SOCS1	3.33	TNFSF13B	2.36
								SOCS3	4.06		
								TLR2	3.12		
								TLR5	3.07		
								TNFSF13B	2.36		
								TRAF5	−2.12		

FC  =  fold change. TREM1 =  triggering receptor expressed on myeloid cells.

### The Transcriptional Response Changes Significantly at 2 Weeks after Treatment Initiation

Since it was observed that the South Africa active TB 664-transcript signature diminished in response to treatment we wished to determine if this was a statistically significant change. To assess this we employed the previously described weighted molecular distance to health (MDTH) algorithm as this generates a quantitative score for the degree of transcriptional perturbation in a disease cohort relative to the controls [16]. Moreover we have already demonstrated that MDTH positively correlates with the severity of active pulmonary TB, as defined by the radiological extent of disease [11]. We found that the median MDTH of the South African untreated active TB 664-transcript signature decreased significantly at 2 weeks onwards, compared to the median pre-treatment MDTH (Figure 2E).

We then developed a novel metric that provides a quantitative measure of an individual’s temporal change in gene expression. This ‘temporal molecular response’ offers a potential advantage in the clinical setting, allowing assessment of each patient’s expression change without reference to a control group. For a given signature the temporal molecular response was determined by measuring the transcriptional perturbation between two time points, and expressing this value as a percentage of the total number of transcripts constituting the signature. The mean temporal molecular response calculated for the active TB 664-transcript signature revealed a statistically significant change in the transcriptional response at 2 weeks after treatment initiation (Figure 2F). This continued to change between 2 weeks and 2 months, and between 2 weeks and 6 months, after treatment initiation (Figure 2F). The magnitude of the patient’s temporal molecular response during treatment (at 2 weeks and 2 months) did not correlate with the magnitude of their untreated transcriptional signature, as measured by MDTH (p<0.01) (Figure S1). This suggests a patient’s untreated transcriptional signature is not predictive of the patient’s treatment response.

In summary we show that this active TB 664-transcript signature (derived from untreated active and latent TB patients) significantly and rapidly changed after 2 weeks of initiating treatment (Figure 2B, E and F).

### A Specific TB Treatment Response Signature Also Significantly Diminishes At 2 Weeks Post Treatment

**Figure 3:**
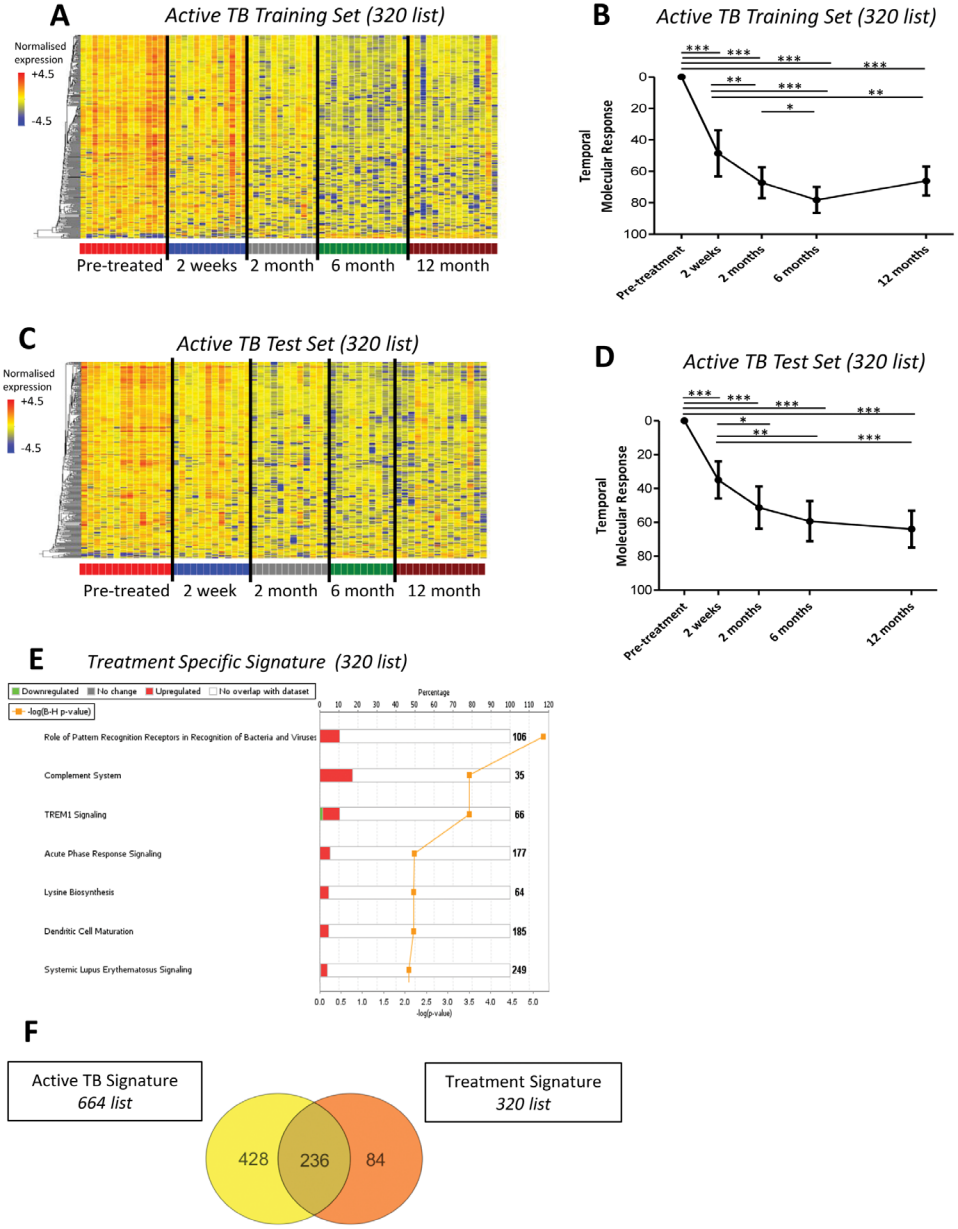
Specific treatment response signature significantly diminishes at 2 weeks onwards. A specific TB treatment response signature was derived from significantly differentially expressed genes between untreated samples in the South Africa Active TB Training Set and their corresponding 6 month samples, 320 transcripts. **(A)** Heatmap of South Africa 2011 Active TB Training Set, normalised to the median of all transcripts, shows transcripts differentiating over time in response to treatment. **(B)** Temporal molecular response further shows significant and early changes in response to TB treatment in the Active TB Training Set (linear mixed models, bars represent mean & 95% confidence intervals, *** = p<0.001, ** = p<0.01, * = p<0.05). **(C)** Heatmap of South Africa 2011 Active TB Test Set, normalised to the median of all transcripts, shows transcripts differentiating over time in response to treatment. **(D)** Temporal molecular response also shows in the Active TB Test Set significant and early changes in response to TB treatment. **(E)** IPA of the 320 transcripts showing the most significant pathways. **(F)** Venn diagram shows many overlapping genes between the active TB 664-transcript signature and the treatment specific 320-signature.

We next sought to define a transcriptional signature that specifically reflected the patients’ response to clinically successful anti-TB treatment (comparing time points 0 and 6 months). To determine this treatment specific signature we first used a computer algorithm to randomise the South Africa 2011 cohort into two groups of patients [12] (Figure 1A). This allowed us to derive the signature from one group of patients (active TB Training Set) and then validate our findings in another independent group of patients (active TB Test Set). 320 transcripts (Table S3) were found to be significantly differentially expressed between the pre-treatment active TB Training Set samples and their paired 6-month treated samples (Figure 3A). The treatment specific 320-transcript signature was shown to rapidly and significantly change at 2 weeks onwards after treatment initiation, in the active TB Training set (Figure 3A and B). This was validated in the active TB Test Set (Figure 3C and D). In both cohorts the change in the temporal molecular response was significant at 2 weeks post-treatment (Figure 3B and D). Analysis of the 320 transcripts by IPA indicated the most significantly represented pathways were related to the innate immune pathways, encompassing genes related to complement and Toll-like receptors (Figure 3E). The treatment specific 320-transcript signature also contained 74% of genes present in the active TB 664-transcript signature (Figure 3F).

Although by applying the temporal molecular response it was observed that the treatment specific 320-transcript signature changed significantly between 2 weeks and 6 months post treatment initiation, this was no longer apparent between 2 months and 6 months post treatment initiation (Figure 3B and D). This could suggest that the transcriptional response reaches a plateau at 2 months and therefore the 2 month gene expression profiles would not be significantly different from the latent TB expression profiles. To establish whether any significant changes occurred between 2 months and the latent TB patients, we compared each of the time points: 2, 6 and 12 months to the latent TB profiles. We determined that 96 transcripts were significantly differentially expressed between 2 months and latent TB (Mann Whitney paired Benjamini Hochberg p<0.01, data not shown). Ingenuity Pathway Analysis demonstrated the top three significant pathways associated with the 96 transcripts were ‘role of NFAT in regulation of the immune response’, ‘integrin signalling’ and ‘primary immunodeficiency signalling’ (data not shown). However no genes were significantly differentially expressed between 6 & 12 months, 6 months & latent TB, and 12 months & latent TB (Mann Whitney paired Benjamini Hochberg p>0.01).

### Measuring an Individual Patient’s Transcriptional Response to anti-TB Treatment

**Figure 4:**
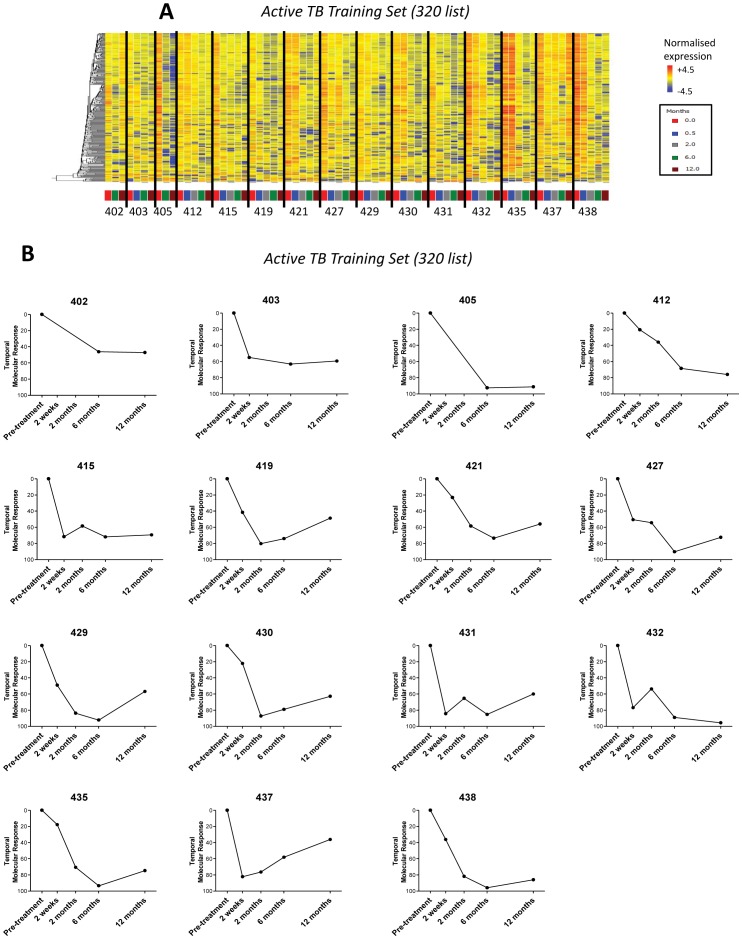
Individual patient’s transcriptional response occurred at a variable rate. 320 gene list, differentially expressed genes derived from comparing the untreated expression profiles and their corresponding end of treatment (6 months) expression profiles in the South Africa 2011 Active TB Training Set. **(A)** Heatmap of South Africa 2011 cohort Active TB Training Set, normalised to the median of all transcripts, shows hierarchical clustered transcripts differentiating over time per individual. **(B)** Each patient’s temporal molecular response diminishes in the Active TB Training Set cohort.

Each patient’s discrete treatment specific response (320 transcripts) is shown in the heatmaps and using the temporal molecular response in Figure 4 and Figure S2. All 29 patients in the active TB treated cohort had a rapid and early positive temporal response after 2 weeks of treatment. Interestingly, not all the individual transcriptional responses were identical (Figure 4A, Figure S2A) as demonstrated by the quantitative scoring provided by the temporal molecular responses (Figure 4B, Figure S2B).

### Validation of the 2 Week Treatment Transcriptional Response

**Figure 5:**
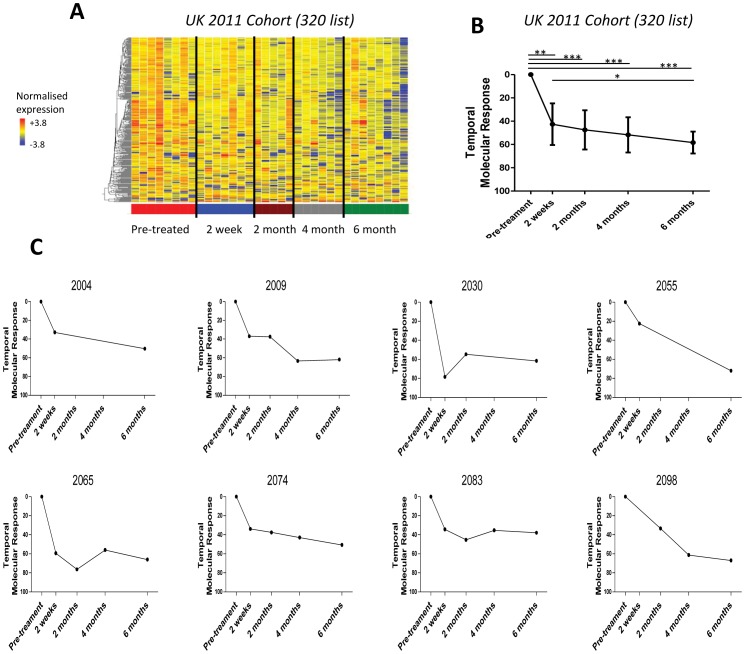
Change in treatment specific signature is validated in an independent UK cohort. 320 gene list derived from the differentially expressed genes between the untreated and 6 month treated samples in the treated South Africa 2011 cohort. **(A)** Heatmap of the treated UK 2011 Cohort, normalised to the median of all transcripts, shows diminution of the treatment specific transcriptional signature in the UK cohort in response to successful anti-TB treatment. **(B)** Temporal molecular response shows significant changes in response at 2 weeks in the UK cohort (linear mixed models, bars represent mean & 95% confidence intervals, *** = p<0.001, ** = p<0.01, * = p<0.05). **(C)** A diminished response can be seen in each patient by their temporal molecular response.

To determine whether the significant change in the treatment specific 320-transcript signature that we had demonstrated in a South African cohort was also applicable to patients in an intermediate burden setting, we tested the signature in a UK cohort. As observed in the South African cohort the signature was rapidly and significantly diminished from 2 weeks post-treatment initiation (Figure 5A and B). The changes in the blood transcriptional response could be clearly quantified in individual patients as shown by the temporal molecular response (Figure 5C). The significant transcriptional blood change correlated with successful treatment of patients as assessed after 6 months by radiographic and clinical parameters (data not shown).

For additional validation that active-TB transcriptional signatures show significant changes as early as 2 weeks after treatment initiation, we demonstrated that the active TB signatures (393- and 86-transcript signatures) from our earlier study [11], also significantly diminished after 2 weeks treatment, in the South Africa 2011 treated cohort (Figure S3A–D).

## Discussion

We derived a whole blood active-TB transcriptional signature consisting of 664 transcripts capable of distinguishing untreated South African active TB patients from South African latent TB patients. We demonstrate that this active-TB transcriptional signature significantly diminishes in active TB patients after just 2 weeks of initiation of clinically successful anti-TB treatment. In addition we demonstrate that a treatment-specific transcriptional signature, consisting of 320 transcripts, derived from comparing a cohort of South African untreated active TB samples to their paired 6-month end of treatment samples, also significantly diminishes after just 2 weeks of anti-TB treatment. Furthermore the significant change in the treatment-specific signature was validated in two more clinically successfully treated cohorts, from the high TB-burden setting of South Africa and from the intermediate TB-burden setting of London, UK. Both the active-TB and treatment-specific transcriptional signatures were dominated by IFN signaling and innate immune response genes. The transcriptional response to anti-TB treatment could also be individually quantified for each patient. Together, these findings suggest that blood transcriptional signatures could be used as early surrogate biomarkers of a successful treatment response, in both the clinical setting and in drug development.

TB treatment monitoring is a major challenge for attempts to eradicate *Mtb* infection. In April 2010 the Centers for Disease Control and National Institutes of Health brought together experts in the field and research scientists with the sole purpose of addressing this problem [17]. Poor treatment monitoring, and hence inadequate treatment, leads to worsening of a patient’s disease, increasing the potential for disease spread and the risk of developing drug resistant mycobacteria. Currently the 2-month sputum culture conversion is the only biomarker of successful TB treatment [6]. However it is time consuming, taking several weeks to grow the bacilli and results can be compromised by contamination. Moreover patients who have clinically improved may be unable to expectorate sputum at 2 months and potentially incorrectly labelled as having a negative culture [18]. Furthermore, although sputum conversion is commonly used as a surrogate end point for treatment response in clinical trials evaluating new drugs, a systematic review and meta-analysis to assess its accuracy in predicting an individual’s treatment failure revealed low sensitivity and only modest specificity [7], [19]. While other biomarkers have also been trialled, including C-reactive protein, IFN-γand neopterin, all have similarly shown poor sensitivity and specificity [20]. Chest X-rays are commonly used in the clinical setting as a marker of treatment response but they generally improve slower than the clinical response and lack specificity as interpretation can be confounded by previous lung damage [18]. Moreover interpretation of radiographic changes in response to treatment has not yet been standardised, and the facilities are not always available in developing countries [8]. Therefore there is clearly a need for early and easily detectable biomarkers for treatment monitoring, capable of potentially identifying poor responses due to drug resistance or lack of treatment adherence, and available for patients unable to produce sputum.

In our earlier study we demonstrated in a small number of patients that blood transcriptional signatures in UK active TB patients diminished after 2 months of anti-TB treatment [11]. In our current study we have shown a significant blood transcriptional response to anti-TB treatment occurs rapidly, as early as 2 weeks (Figures 2–5, E2–3). This early transcriptional response could be as a consequence of the observed rapid and high killing capacity of antimycobacterial antibiotics leading to a substantial reduction in mycobacterial load [21], [22], [23]. Although the signatures we have derived may not be completely specific for active TB, since clinically similar diseases such as sarcoidosis show common transcripts [24], demonstration of a response to antimycobacterial therapy as we show here, could help resolve this overlap and so improve diagnostic specificity.

We have shown here that the whole blood active-TB transcriptional signature is dominated by IFN signaling and innate immune response genes. These findings are in agreement with our previous work [11], and with other gene expression studies in TB [25], [26]. This robust correlation occurring between different host populations, likely different *Mtb* strains, diverse environments and microarray analysis strategies indicates that blood transcriptomics have great potential to be developed into robust novel diagnostic tools. Furthermore we demonstrate here that the derived treatment specific 320-transcript signature also had many genes in common with the active TB 664-transcript signature (Figure 3F). This overlap of genes is highly suggestive that this study will help guide future development of a subset of genes that most accurately correlates with a patient’s response to anti-TB treatment, acting as a surrogate marker of treatment failure or success.

Due to the ethical design of this study we do not present active TB patients who did not respond to TB treatment. Our study has demonstrated a very important proof-of-principle that active TB patients who are successfully treated have a dramatic measurable change in their blood gene expression profiles as early as 2 weeks. The use of a commercially available whole genome microarray platform together with broadly available bioinformatics analyses programmes will easily allow rapid validation in subsequent TB treatment studies, including a comparison with patients with MDR-TB and HIV/TB co-infected cohorts. This study focussed on TB patients who are not co-infected with HIV, as they represent the majority of patients infected with *Mtb*. WHO 2010 reports that of the 1.4 million deaths, three-quarters were not known to be co-infected with HIV [2].

To the best of our knowledge no other studies have specifically derived transcriptional signatures of response to TB treatment. However two other studies have described relevant treatment related transcriptional differences. Mistry *et al* found that patients who had completed a course of anti-TB treatment displayed similar expression profiles to a latent TB group, but they did not examine any patients during their anti-TB treatment course, and used custom arrays [27], therefore more difficult for others to validate. Joosten *et al* showed in a small number of samples that their active TB gene set diminished after 2 months of anti-TB treatment, however they did not examine any patients at earlier timepoints [28]. Our early TB treatment blood transcriptional signature has great potential for development as blood biomarkers for clinical use and could be measured in the field using a polymerase chain reaction assay, similar to the WHO endorsed GeneXpert MTB/RIF test already in use for TB diagnostics in both developing and developed countries. However a blood host biomarker, based on our transcriptional signature, would have advantages over the GeneXpert test since it would not require sputum.

A further problem in the management of TB is the extended length of treatment, requiring a minimum duration of 6 months. However the treatment duration required for maximum efficacy and preventing resistance, has not been fully established. The ability therefore to stratify patients into groups requiring shorter or longer treatment durations, particularly in resource limited settings, could be of value in improving patient compliance and reducing treatment related side effects. We demonstrate here that some patient’s transcriptional response appeared to plateau before 6 months (Figure 4 and Figure S2 **)** suggesting blood transcriptional signatures may help develop personalized treatment regimes.

In summary we have shown that transcriptional signatures, measured in easily accessible whole blood, showed a significant response to anti-TB treatment, as early as 2 weeks after initiation of treatment, far quicker than currently available tests. In addition, we have demonstrated this early response to anti-TB treatment occurs in both high- and intermediate-burden settings. The transcriptional response could be measured for each individual TB patient, thus providing a potential clinical tool for single patient treatment monitoring. Furthermore this could aid in patient stratification for treatment with differing regimen lengths. These findings provide a strong foundation for the development of an early biomarker of successful anti-TB treatment response. Development will require validation of these findings in larger cohorts, including a group of patients who fail to respond to anti-TB treatment. This potential biomarker of early treatment response could allow rapid detection of inadequate treatment regimens and poor treatment compliance, therefore ultimately reducing disease spread and drug resistant *Mtb*.

## Supporting Information

Figure S1 **The Changing Transcriptional Response Is Independent of the Magnitude of the Untreated Transcriptional Signature.** Weighted molecular distance to health (MDTH) has been shown to correlate with radiological extent of active TB disease [11]. The magnitude of the patient’s temporal molecular response during treatment, at both 2 weeks and 2 months, did not correlate with the magnitude of their untreated transcriptional signature, as evidenced measured by MDTH (linear regression r^2^<0.25, p>0.01). However, the patient’s temporal molecular response after treatment, at 6 months and 12 months, did significantly correlate with their untreated MDTH (linear regression r^2^ = 0.32, p = 0.003 and r^2^ = 0.38, p = 0.0004 respectively).
(TIF)

Figure S2 **Individual Patient’s Transcriptional Response Occurred at a Variable Rate.** 320 gene list, differentially expressed genes derived from comparing the untreated expression profiles and their corresponding end of treatment (6 months) expression profiles in the South Africa 2011 Active TB Training Set. **(A)** Heatmap of South Africa 2011 cohort Active TB Test Set shows hierarchical clustered transcripts normalised to the median of all transcripts, differentiating over time per individual. **(B)** Each patient’s temporal molecular response in the South Africa 2011 cohort Active TB Test Set.
(TIF)

Figure S3 **The Berry** ***et al*** **Active TB Signatures Also Significantly Diminish in Response to Successful Treatment.** 393 and 86 signatures were defined as described [9] differentiating active TB patients from latent TB patients/healthy controls (393 signature), and differentiating active TB patients from patients with other inflammatory and infectious diseases (86 signature). Both signatures diminished in response to anti-TB treatment in the treated South Africa 2011 cohort. **(A)** Heatmap shows hierarchical clustering of the transcripts, normalised to the median of all transcripts, with samples grouped into time points. **(B)** Heatmap shows hierarchical clustering of the transcripts, normalised to the median of all transcripts, with samples grouped per individual. **(C)** Temporal molecular response further shows significant and early changes in response to anti-TB treatment (linear mixed models, bars represent mean & 95% confidence intervals, *** = p<0.001, ** = p<0.01, * = p<0.05). Summary of demographics and clinical data. **(1A)** South Africa 2011 cohort. Of the 29 untreated active TB patients, 16 were also included in our previous Berry *et al* study [11]. Of the 38 untreated latent TB patients, 17 were also included in our previous Berry *et al* study [11]. For this present study all untreated samples were processed again alongside all the other samples. **(1B)** UK 2011 cohort.
(TIF)

Table S1 **Summary of demographics and clinical data.** **(1A)** South Africa 2011 cohort. Of the 29 untreated active TB patients, 16 were also included in our previous Berry *et al* study [11]. Of the 38 untreated latent TB patients, 17 were also included in our previous Berry *et al* study [11]. For this present study all untreated samples were processed again alongside all the other samples. **(1B)** UK 2011 cohort.
(XLSX)

Table S2 **List of the 664 transcripts in the active-TB transcriptional signature.**
(XLSX)

Table S3 **List of the 320 transcripts in the treatment-specific transcriptional signature.**
(XLSX)
