# Supplementary figures and images for: Detectable Changes in The Blood Transcriptome Are Present after Two Weeks of Antituberculosis Therapy
Source: PLoS One. 2012 Oct 2;7(10):e46191. doi: 10.1371/journal.pone.0046191 (PMC3462772; doi:10.1371/journal.pone.0046191)

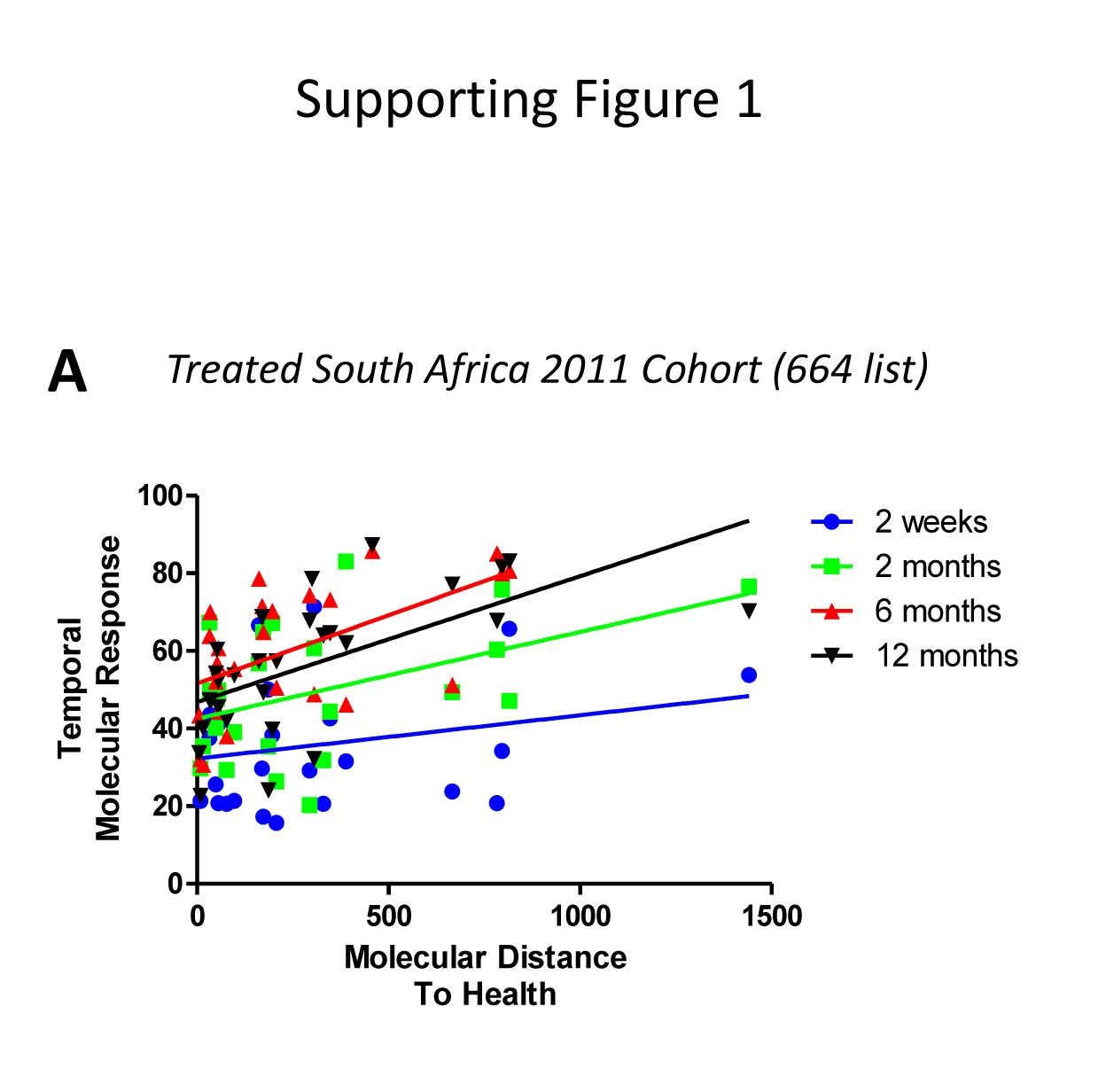

Supplement: Figure S1 — The Changing Transcriptional Response Is Independent of the Magnitude of the Untreated Transcriptional Signature. Weighted molecular distance to health (MDTH) has been shown to correlate with radiological extent of active TB disease [11]. The magnitude of the patient’s temporal molecular response during treatment, at both 2 weeks and 2 months, did not correlate with the magnitude of their untreated transcriptional signature, as evidenced measured by MDTH (linear regression r2<0.25, p>0.01). However, the patient’s temporal molecular response after treatment, at 6 months and 12 months, did significantly correlate with their untreated MDTH (linear regression r2 = 0.32, p = 0.003 and r2 = 0.38, p = 0.0004 respectively). (TIF) [file pone.0046191.s001.tif]

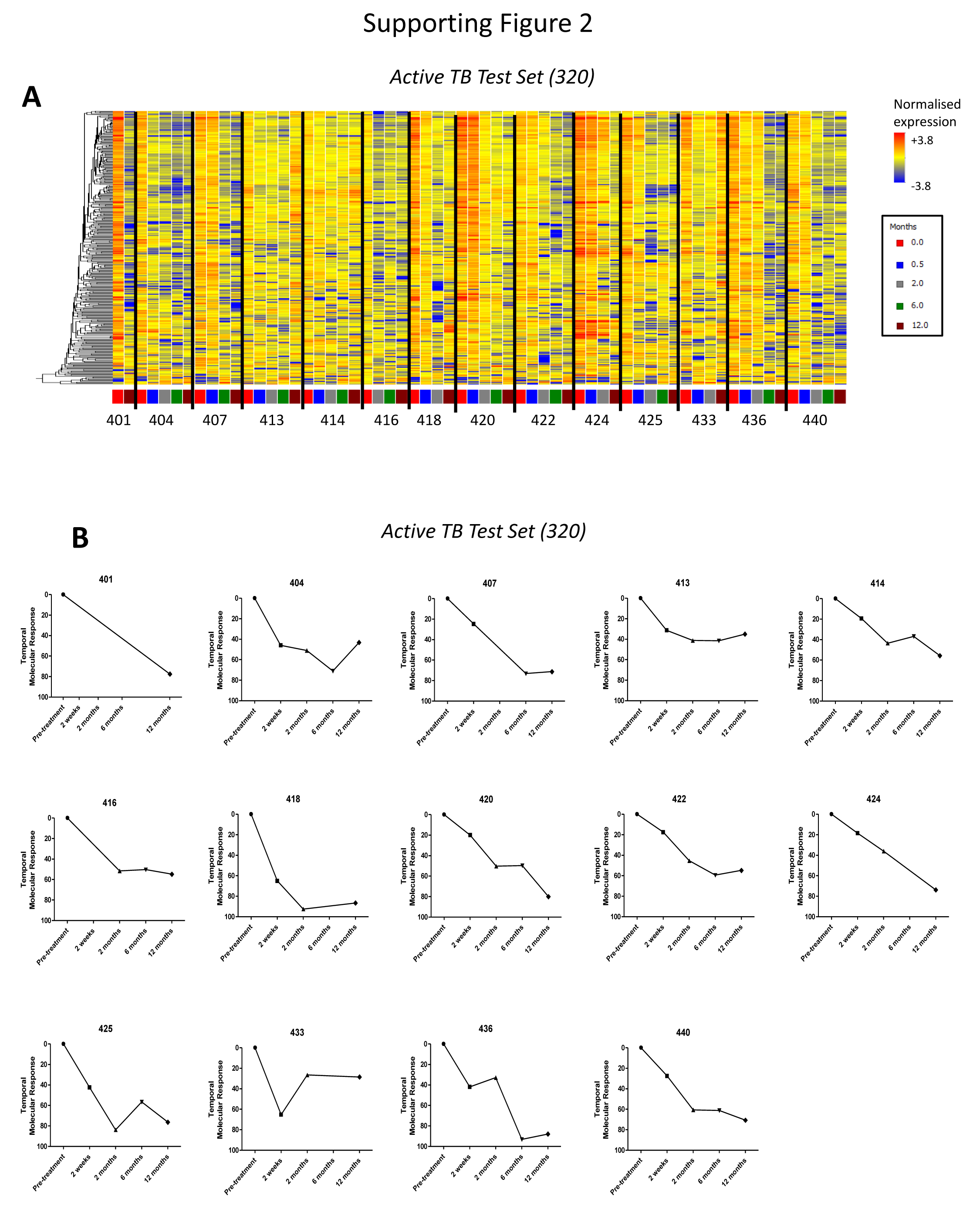

Supplement: Figure S2 — Individual Patient’s Transcriptional Response Occurred at a Variable Rate. 320 gene list, differentially expressed genes derived from comparing the untreated expression profiles and their corresponding end of treatment (6 months) expression profiles in the South Africa 2011 Active TB Training Set. (A) Heatmap of South Africa 2011 cohort Active TB Test Set shows hierarchical clustered transcripts normalised to the median of all transcripts, differentiating over time per individual. (B) Each patient’s temporal molecular response in the South Africa 2011 cohort Active TB Test Set. (TIF) [file pone.0046191.s002.tif]

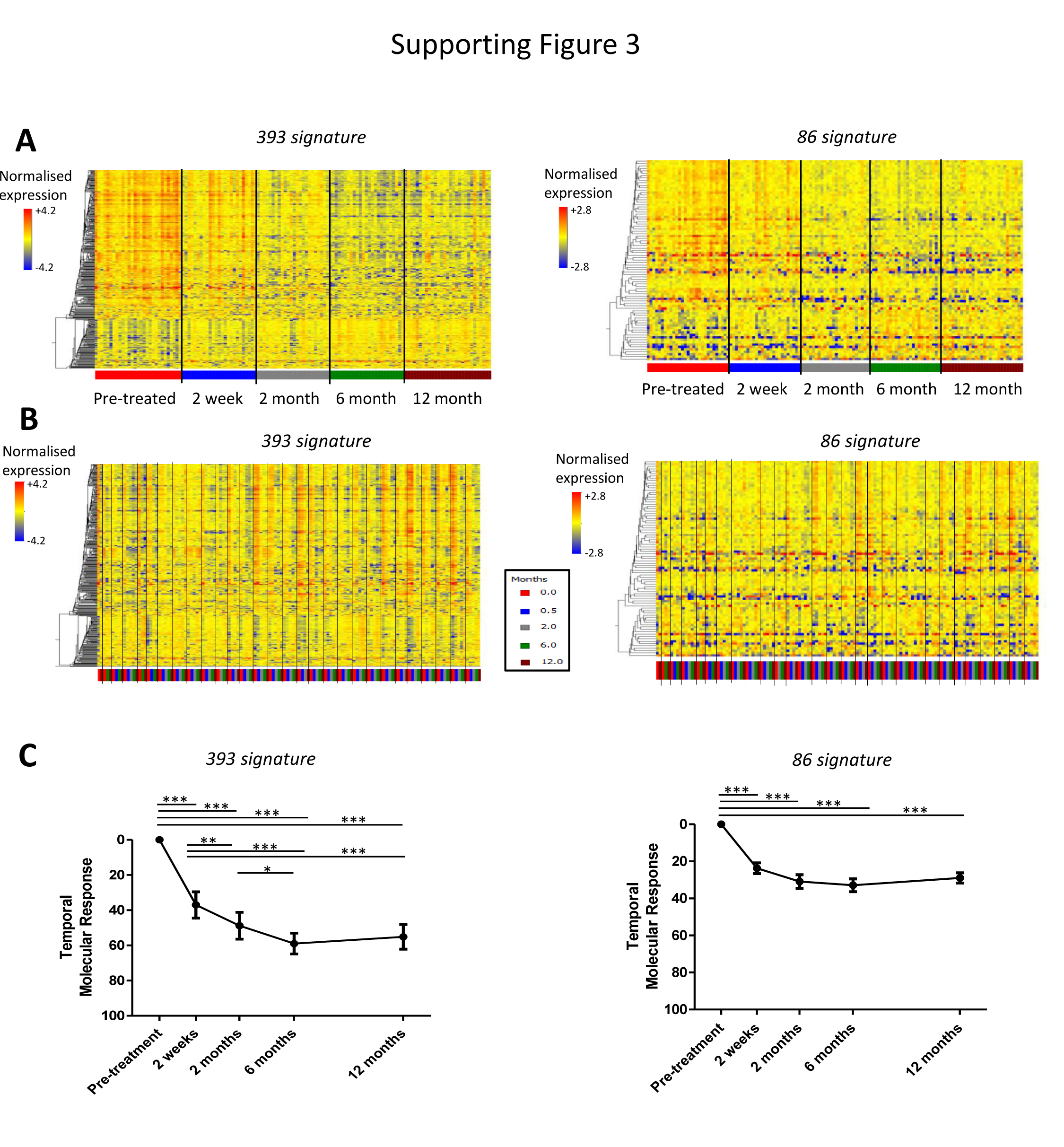

Supplement: Figure S3 — The Berry et al Active TB Signatures Also Significantly Diminish in Response to Successful Treatment. 393 and 86 signatures were defined as described [9] differentiating active TB patients from latent TB patients/healthy controls (393 signature), and differentiating active TB patients from patients with other inflammatory and infectious diseases (86 signature). Both signatures diminished in response to anti-TB treatment in the treated South Africa 2011 cohort. (A) Heatmap shows hierarchical clustering of the transcripts, normalised to the median of all transcripts, with samples grouped into time points. (B) Heatmap shows hierarchical clustering of the transcripts, normalised to the median of all transcripts, with samples grouped per individual. (C) Temporal molecular response further shows significant and early changes in response to anti-TB treatment (linear mixed models, bars represent mean & 95% confidence intervals, *** = p<0.001, ** = p<0.01, * = p<0.05). Summary of demographics and clinical data. (1A) South Africa 2011 cohort. Of the 29 untreated active TB patients, 16 were also included in our previous Berry et al study [11]. Of the 38 untreated latent TB patients, 17 were also included in our previous Berry et al study [11]. For this present study all untreated samples were processed again alongside all the other samples. (1B) UK 2011 cohort. (TIF) [file pone.0046191.s003.tif]
